# 1′′-Allyl-5′′-(4-meth­oxy­benzyl­idene)-7′-(4-methoxy­phen­yl)-1′,3′,5′,6′,7′,7a′-hexa­hydro­dispiro­[ace­naphthyl­ene-1,5′-pyrrolo­[1,2-*c*][1,3]thia­zole-6′,3′′-piperidine]-2,4′′(1*H*)-dione

**DOI:** 10.1107/S1600536813020084

**Published:** 2013-07-27

**Authors:** Abulrahman I. Almansour, Raju Suresh Kumar, Natarajan Arumugam, R. A. Nagalakshmi, J. Suresh

**Affiliations:** a Department of Chemistry, College of Sciences, King Saud University, PO Box 2455, Riyadh 11451, Saudi Arabia; b Department of Physics, The Madura College, Madurai 625 011, India

## Abstract

In the title compound, C_39_H_36_N_2_O_4_S, the piperidine ring adopts a twisted half-chair conformation. In the pyrrolo­thia­zole fused-ring system, the pyrrole ring adopts an envelope conformation (with the C atom bound to the thia­zole ring being the flap atom) and the thia­zole ring also exhibits an envelope conformation (with the N atom bound to the pyrrole ring as the flap). The mol­ecular structure features a weak intra­molecular C—H⋯O inter­action. In the crystal, a C—H⋯O inter­action forms a linear chain along the diagonal of the *ac* plane, generating a *C*(14) graph-set motif. A weak C—H⋯π inter­action also occurs.

## Related literature

For the importance of hetrocyclic rings, see: Guengerich *et al.* (1973 ▶); Lalezari & Schwartz (1988 ▶); Tsuge & Kanemasa (1989 ▶); Puder *et al.* (2000 ▶); Nair & Suja (2007 ▶). For related ace­naphthyl­ene structures, see: Suresh *et al.* (2011 ▶). For additional conformation analysis, see: Cremer & Pople (1975 ▶). For hydrogen-bond motifs, see: Bernstein *et al.* (1995 ▶).
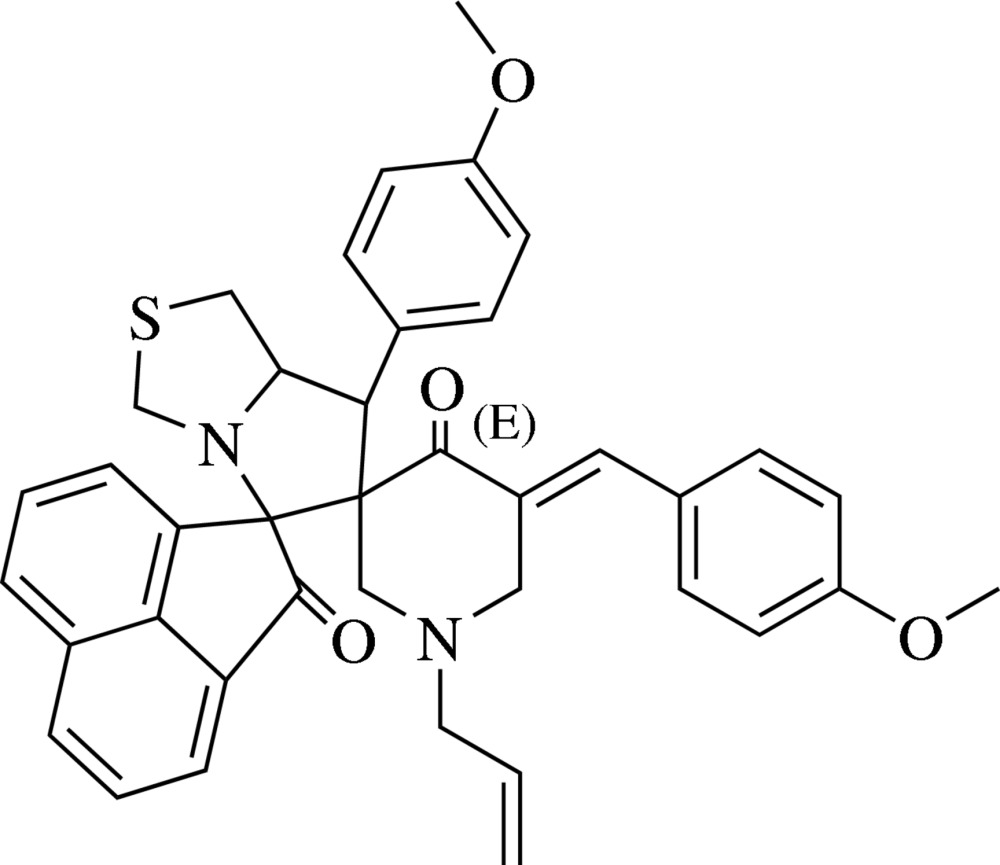


## Experimental

### 

#### Crystal data

C_39_H_36_N_2_O_4_S
*M* *_r_* = 628.76
Monoclinic, 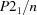

*a* = 11.3956 (7) Å
*b* = 20.1346 (12) Å
*c* = 14.3860 (8) Å
β = 103.153 (1)°
*V* = 3214.2 (3) Å^3^
*Z* = 4
Mo *K*α radiation
μ = 0.15 mm^−1^
*T* = 293 K
0.21 × 0.19 × 0.18 mm

#### Data collection

Bruker Kappa APEXII diffractometer
Absorption correction: multi-scan (*SADABS*; Sheldrick, 1996 ▶) *T*_min_ = 0.967, *T*_max_ = 0.974
26225 measured reflections
5979 independent reflections
4873 reflections with *I* > 2σ(*I*)
*R*_int_ = 0.024

#### Refinement

*R*[*F* ^2^ > 2σ(*F* ^2^)] = 0.037
*wR*(*F* ^2^) = 0.104
*S* = 1.07
5979 reflections
415 parameters
H-atom parameters constrained
Δρ_max_ = 0.29 e Å^−3^
Δρ_min_ = −0.27 e Å^−3^

### 

Data collection: *APEX2* (Bruker, 2004 ▶); cell refinement: *SAINT* (Bruker, 2004 ▶); data reduction: *SAINT*; program(s) used to solve structure: *SHELXS97* (Sheldrick, 2008 ▶); program(s) used to refine structure: *SHELXL97* (Sheldrick, 2008 ▶); molecular graphics: *PLATON* (Spek, 2009 ▶); software used to prepare material for publication: *SHELXL97*.

## Supplementary Material

Crystal structure: contains datablock(s) global, I. DOI: 10.1107/S1600536813020084/gw2135sup1.cif

Structure factors: contains datablock(s) I. DOI: 10.1107/S1600536813020084/gw2135Isup2.hkl

Additional supplementary materials:  crystallographic information; 3D view; checkCIF report

## Figures and Tables

**Table 1:** Hydrogen-bond geometry (Å, °) *Cg*1 is the centroid of the C52–C57 ring.

*D*—H⋯*A*	*D*—H	H⋯*A*	*D*⋯*A*	*D*—H⋯*A*
C2—H2*B*⋯O3	0.97	2.45	2.9587 (19)	113
C93—H93⋯O1^i^	0.93	2.49	3.343 (2)	153
C58—H58*B*⋯*Cg*1^ii^	0.96	2.85	3.487 (2)	125

Symmetry codes: (i) 
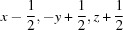
; (ii) 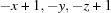.

**Fig. 1.**
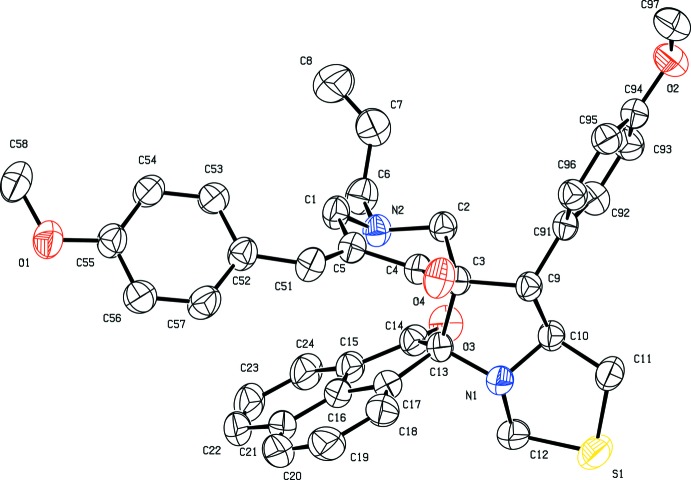
The molecular structure of (I), showing 20% probability displacement ellipsoids and the atom-numbering scheme. H-atoms are omitted for clarity.

**Fig. 2.**
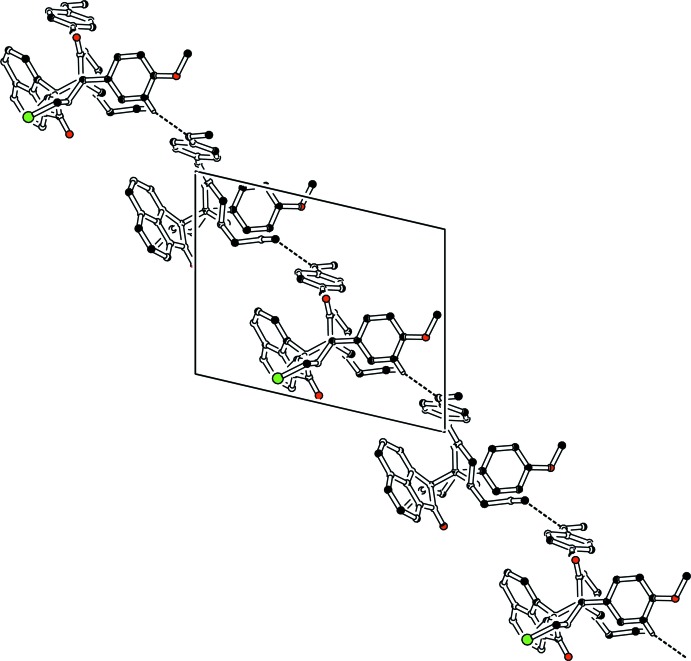
The partial packing diagram showing C—H···O interactions.

## supplementary crystallographic information

### Comment

[3 + 2]-cycloaddition of azomethine ylides to olefinic dipolarophiles constitutes a facile approach for the construction of five membered heterocyclic rings of biological importance (Tsuge & Kanemasa, 1989; Nair & Suja, 2007). Among these heterocycles, pyrrolo[2,1-*b*]thiazole is an unusual ring system with a ntineoplastic (Lalezari & Schwartz, 1988) activities. Piperidine ring systems are of immense interest in the pharmaceutical industry as they exhibit a wide range of biological activities (Guengerich *et al.*, 1973; Puder *et al.*, 2000). In view of its medicinal importance and in conjunction with our research interests, we synthesized the title compound and report here its X-ray structure.

In the title compound (Fig.1) C_39_H_36_N_2_O_2_S, the piperidine ring adopts a twisted half chair conformation with atoms N2 and C2 deviating by -0.1919 (1) Å and -0.5644 (1) Å respectively from the least squares plane defined by other atoms (C1/C3/C4/C5). In the fused system, the thiazole ring adopts an envelope conformation with N1 atom as a flap atom, displaced by a -0.6085 (1) Å from the mean plane through the remaining atoms and the pyrrole ring adopts an envelope conformation with C10 atom as a flap atom, displaced by a 0.6545 (1) Å from the mean plane through the remaining atoms. The methyl group of the methoxy phenyl rings are in equtorial orientations as evidenced from the torsion angles C58—O1—C55—C56 = 172.59 (16) ° and C97—O2—C94—C93 = 172.07 (14) °. The methoxy phenyl rings are oriented at angles of 56.25 (1) ° (C52—C57) and 86.90 (1) ° (C91—C97) with the mean plane of the piperidine ring. The twist of the methoxyphenyl ring attached to C51 may be due to the non-bonded interactions between one of the *ortho* H atoms of the aryl ring and equtorial H atoms at the 2 position of the piperidine ring. The C—C bond lengths and C—C—C angles in the acenaphthylene group compare with those of related structure (Suresh *et al.*,2011).

The structure features a weak intra-molecular interaction. An inter-molecular C—H···O interaction forms a linear chain along the diagonal of the *ac* plane generating a graph set motif of C_1_^1^(14) (Bernstein *et al.*,1995). In addition a weak C—H···π interaction (Table 1) is also observed.

### Experimental

A mixture of (3E,5E)-3,5-bis(4-methoxyphenylmethylidene)-1-allylpiperidin-4-one (1 mmol), acenaphthenequinone (1 mmol) and 1,3-thiazolane-4-carboxylic acid (1 mmol) was dissolved in methanol (10 ml) and refluxed for 1 h. After completion of the reaction as evident from TLC, the mixture was poured into water (50 ml), the precipitated solid was filtered and washed with water (100 ml) to obtain the product as pale yellow solid. Melting point = 412–414 K; Yield = 94%

### Refinement

H atoms were placed at calculated positions and allowed to ride on their carrier atoms with C—H = 0.93–0.98 Å. *U*_iso_ = 1.2*U*_eq_(C) for CH_2_ and CH groups and *U*_iso_ = 1.5*U*_eq_(C) for CH_3_ group.

### Crystal data

**Table:**

C_39_H_36_N_2_O_4_S	*F*(000) = 1328
*M**_r_* = 628.76	*D*_x_ = 1.299 Mg m^−^^3^
Monoclinic, *P*2_1_/*n*	Mo *K*α radiation, λ = 0.71073 Å
Hall symbol: -P 2yn	Cell parameters from 2000 reflections
*a* = 11.3956 (7) Å	θ = 2–31°
*b* = 20.1346 (12) Å	µ = 0.15 mm^−^^1^
*c* = 14.3860 (8) Å	*T* = 293 K
β = 103.153 (1)°	Block, yellow
*V* = 3214.2 (3) Å^3^	0.21 × 0.19 × 0.18 mm
*Z* = 4	

### Data collection

**Table:**

Bruker Kappa APEXII diffractometer	5979 independent reflections
Radiation source: fine-focus sealed tube	4873 reflections with *I* > 2σ(*I*)
Graphite monochromator	*R*_int_ = 0.024
Detector resolution: 0 pixels mm^-1^	θ_max_ = 25.5°, θ_min_ = 2.0°
ω and φ scans	*h* = −13→13
Absorption correction: multi-scan (*SADABS*; Sheldrick, 1996)	*k* = −24→23
*T*_min_ = 0.967, *T*_max_ = 0.974	*l* = −17→17
26225 measured reflections	

### Refinement

**Table:**

Refinement on *F*^2^	Primary atom site location: structure-invariant direct methods
Least-squares matrix: full	Secondary atom site location: difference Fourier map
*R*[*F*^2^ > 2σ(*F*^2^)] = 0.037	Hydrogen site location: inferred from neighbouring sites
*wR*(*F*^2^) = 0.104	H-atom parameters constrained
*S* = 1.07	*w* = 1/[σ^2^(*F*_o_^2^) + (0.0448*P*)^2^ + 0.8433*P*] where *P* = (*F*_o_^2^ + 2*F*_c_^2^)/3
5979 reflections	(Δ/σ)_max_ < 0.001
415 parameters	Δρ_max_ = 0.29 e Å^−^^3^
0 restraints	Δρ_min_ = −0.27 e Å^−^^3^

### Special details

**Table:**

Geometry. All e.s.d.'s (except the e.s.d. in the dihedral angle between two l.s. planes) are estimated using the full covariance matrix. The cell e.s.d.'s are taken into account individually in the estimation of e.s.d.'s in distances, angles and torsion angles; correlations between e.s.d.'s in cell parameters are only used when they are defined by crystal symmetry. An approximate (isotropic) treatment of cell e.s.d.'s is used for estimating e.s.d.'s involving l.s. planes.
Refinement. Refinement of *F*^2^ against ALL reflections. The weighted *R*- factor *wR* and goodness of fit *S* are based on *F*^2^, conventional *R*-factors *R* are based on *F*, with *F* set to zero for negative *F*^2^. The threshold expression of *F^2^* > σ(*F*^2^) is used only for calculating *R*- factors (gt) *etc*. and is not relevant to the choice of reflections for refinement. *R*-factors based on *F*^2^ are statistically about twice as large as those based on *F*, and *R*- factors based on ALL data will be even larger.

### Fractional atomic coordinates and isotropic or equivalent isotropic displacement parameters (Å^2^)

**Table:**

	*x*	*y*	*z*	*U*_iso_*/*U*_eq_	
C1	0.38453 (15)	0.24423 (8)	0.61103 (12)	0.0465 (4)	
H1A	0.3731	0.1988	0.5882	0.056*	
H1B	0.4268	0.2429	0.6776	0.056*	
C2	0.27822 (14)	0.34635 (7)	0.62444 (11)	0.0406 (3)	
H2A	0.3379	0.3536	0.6834	0.049*	
H2B	0.2018	0.3640	0.6320	0.049*	
C3	0.31686 (13)	0.38124 (7)	0.54151 (10)	0.0376 (3)	
C4	0.44181 (13)	0.35396 (7)	0.53899 (11)	0.0409 (3)	
C5	0.46034 (14)	0.28104 (7)	0.55445 (11)	0.0430 (3)	
C6	0.19271 (16)	0.23989 (9)	0.65863 (13)	0.0536 (4)	
H6A	0.1854	0.1940	0.6377	0.064*	
H6B	0.1125	0.2590	0.6438	0.064*	
C7	0.23879 (18)	0.24090 (10)	0.76404 (14)	0.0617 (5)	
H7	0.2569	0.2823	0.7922	0.074*	
C8	0.2563 (2)	0.19024 (12)	0.82035 (18)	0.0892 (7)	
H8A	0.2396	0.1477	0.7957	0.107*	
H8B	0.2855	0.1962	0.8856	0.107*	
C9	0.31827 (13)	0.45865 (7)	0.54978 (10)	0.0387 (3)	
H9	0.3782	0.4748	0.5160	0.046*	
C10	0.19504 (14)	0.47781 (7)	0.48948 (11)	0.0417 (3)	
H10	0.1316	0.4678	0.5233	0.050*	
C11	0.17877 (16)	0.54723 (8)	0.44674 (13)	0.0532 (4)	
H11A	0.2551	0.5650	0.4390	0.064*	
H11B	0.1456	0.5770	0.4872	0.064*	
C12	0.07720 (16)	0.44611 (9)	0.33773 (12)	0.0545 (4)	
H12A	0.0076	0.4290	0.3582	0.065*	
H12B	0.0802	0.4264	0.2768	0.065*	
C13	0.22302 (13)	0.36619 (7)	0.44231 (10)	0.0404 (3)	
C14	0.11388 (14)	0.32178 (8)	0.45544 (11)	0.0449 (4)	
C15	0.11378 (15)	0.26054 (8)	0.39828 (12)	0.0482 (4)	
C16	0.20475 (15)	0.26712 (8)	0.34768 (11)	0.0465 (4)	
C17	0.26952 (15)	0.32703 (8)	0.36735 (11)	0.0438 (3)	
C18	0.35670 (17)	0.34039 (9)	0.31920 (12)	0.0551 (4)	
H18	0.4003	0.3798	0.3299	0.066*	
C19	0.3800 (2)	0.29318 (11)	0.25234 (13)	0.0674 (5)	
H19	0.4395	0.3025	0.2194	0.081*	
C20	0.3185 (2)	0.23472 (10)	0.23449 (13)	0.0688 (6)	
H20	0.3373	0.2048	0.1908	0.083*	
C21	0.22627 (18)	0.21948 (8)	0.28235 (12)	0.0570 (5)	
C22	0.1515 (2)	0.16223 (9)	0.27252 (14)	0.0697 (6)	
H22	0.1620	0.1287	0.2307	0.084*	
C23	0.0649 (2)	0.15566 (9)	0.32311 (16)	0.0711 (6)	
H23	0.0186	0.1172	0.3156	0.085*	
C24	0.04265 (17)	0.20495 (9)	0.38639 (14)	0.0606 (5)	
H24	−0.0184	0.1999	0.4191	0.073*	
C51	0.53982 (15)	0.25243 (8)	0.51160 (12)	0.0492 (4)	
H51	0.5801	0.2812	0.4790	0.059*	
C52	0.57272 (15)	0.18221 (8)	0.50855 (12)	0.0484 (4)	
C53	0.58985 (16)	0.13945 (9)	0.58581 (12)	0.0550 (4)	
H53	0.5803	0.1555	0.6443	0.066*	
C54	0.62084 (17)	0.07332 (9)	0.57851 (12)	0.0550 (4)	
H54	0.6328	0.0457	0.6317	0.066*	
C55	0.63385 (15)	0.04874 (8)	0.49180 (12)	0.0510 (4)	
C56	0.61851 (18)	0.09096 (10)	0.41350 (13)	0.0621 (5)	
H56	0.6277	0.0748	0.3550	0.074*	
C57	0.58980 (17)	0.15644 (9)	0.42261 (13)	0.0587 (5)	
H57	0.5815	0.1844	0.3701	0.070*	
C58	0.6933 (2)	−0.05957 (10)	0.55429 (16)	0.0698 (5)	
H58A	0.7101	−0.1027	0.5321	0.105*	
H58B	0.6274	−0.0626	0.5854	0.105*	
H58C	0.7634	−0.0432	0.5986	0.105*	
C91	0.35268 (14)	0.48671 (7)	0.65013 (10)	0.0393 (3)	
C92	0.26940 (15)	0.49766 (8)	0.70580 (12)	0.0495 (4)	
H92	0.1890	0.4866	0.6817	0.059*	
C93	0.30398 (16)	0.52474 (9)	0.79634 (12)	0.0536 (4)	
H93	0.2466	0.5318	0.8321	0.064*	
C94	0.42242 (16)	0.54125 (8)	0.83392 (11)	0.0470 (4)	
C95	0.50713 (15)	0.53016 (8)	0.78109 (11)	0.0469 (4)	
H95	0.5877	0.5405	0.8062	0.056*	
C96	0.47143 (14)	0.50337 (7)	0.68956 (11)	0.0436 (3)	
H96	0.5291	0.4965	0.6540	0.052*	
C97	0.56944 (19)	0.57887 (11)	0.96906 (14)	0.0698 (5)	
H97A	0.5751	0.5985	1.0308	0.105*	
H97B	0.6063	0.6078	0.9309	0.105*	
H97C	0.6102	0.5368	0.9763	0.105*	
N1	0.18725 (12)	0.43348 (6)	0.40858 (9)	0.0439 (3)	
N2	0.26677 (11)	0.27556 (6)	0.60308 (9)	0.0431 (3)	
O1	0.66222 (13)	−0.01537 (6)	0.47538 (10)	0.0665 (4)	
O2	0.44643 (12)	0.56933 (6)	0.92363 (9)	0.0633 (3)	
O3	0.03616 (10)	0.34017 (6)	0.49502 (9)	0.0554 (3)	
O4	0.52104 (10)	0.38980 (6)	0.52376 (9)	0.0555 (3)	
S1	0.07354 (5)	0.53678 (2)	0.33048 (4)	0.06657 (16)	

### Atomic displacement parameters (Å^2^)

**Table:**

	*U*^11^	*U*^22^	*U*^33^	*U*^12^	*U*^13^	*U*^23^
C1	0.0554 (9)	0.0336 (8)	0.0529 (9)	0.0000 (7)	0.0174 (8)	0.0018 (7)
C2	0.0422 (8)	0.0368 (8)	0.0434 (8)	−0.0017 (6)	0.0109 (6)	0.0005 (6)
C3	0.0391 (7)	0.0334 (7)	0.0405 (8)	−0.0029 (6)	0.0096 (6)	−0.0004 (6)
C4	0.0429 (8)	0.0379 (8)	0.0420 (8)	−0.0020 (6)	0.0096 (6)	0.0003 (6)
C5	0.0432 (8)	0.0387 (8)	0.0466 (8)	0.0007 (6)	0.0091 (7)	0.0008 (6)
C6	0.0536 (10)	0.0450 (9)	0.0655 (11)	−0.0081 (8)	0.0202 (8)	0.0055 (8)
C7	0.0734 (12)	0.0547 (11)	0.0649 (11)	−0.0112 (9)	0.0322 (10)	−0.0002 (9)
C8	0.117 (2)	0.0724 (15)	0.0745 (14)	−0.0065 (14)	0.0144 (14)	0.0113 (12)
C9	0.0414 (8)	0.0326 (7)	0.0427 (8)	−0.0038 (6)	0.0111 (6)	−0.0001 (6)
C10	0.0442 (8)	0.0354 (8)	0.0453 (8)	−0.0010 (6)	0.0095 (7)	−0.0015 (6)
C11	0.0555 (10)	0.0388 (9)	0.0611 (10)	0.0031 (7)	0.0045 (8)	0.0034 (7)
C12	0.0596 (10)	0.0515 (10)	0.0472 (9)	0.0011 (8)	0.0011 (8)	0.0009 (7)
C13	0.0440 (8)	0.0337 (8)	0.0430 (8)	−0.0029 (6)	0.0087 (6)	−0.0014 (6)
C14	0.0440 (8)	0.0407 (8)	0.0478 (9)	−0.0025 (7)	0.0055 (7)	0.0012 (7)
C15	0.0509 (9)	0.0379 (8)	0.0498 (9)	−0.0026 (7)	−0.0009 (7)	0.0005 (7)
C16	0.0544 (9)	0.0375 (8)	0.0423 (8)	0.0051 (7)	−0.0001 (7)	−0.0001 (6)
C17	0.0505 (9)	0.0399 (8)	0.0395 (8)	0.0021 (7)	0.0072 (7)	0.0003 (6)
C18	0.0643 (11)	0.0548 (10)	0.0488 (9)	0.0009 (8)	0.0180 (8)	0.0008 (8)
C19	0.0779 (13)	0.0771 (14)	0.0520 (10)	0.0158 (11)	0.0250 (10)	0.0028 (10)
C20	0.0914 (15)	0.0627 (12)	0.0506 (10)	0.0249 (11)	0.0125 (10)	−0.0084 (9)
C21	0.0739 (12)	0.0434 (9)	0.0460 (9)	0.0142 (8)	−0.0022 (8)	−0.0042 (7)
C22	0.0955 (15)	0.0405 (10)	0.0593 (11)	0.0111 (10)	−0.0115 (11)	−0.0112 (8)
C23	0.0808 (14)	0.0382 (10)	0.0795 (14)	−0.0076 (9)	−0.0122 (12)	−0.0028 (9)
C24	0.0613 (11)	0.0429 (9)	0.0693 (12)	−0.0098 (8)	−0.0023 (9)	0.0015 (8)
C51	0.0485 (9)	0.0439 (9)	0.0577 (10)	0.0040 (7)	0.0173 (8)	0.0070 (7)
C52	0.0483 (9)	0.0452 (9)	0.0549 (9)	0.0072 (7)	0.0186 (7)	0.0043 (7)
C53	0.0671 (11)	0.0534 (10)	0.0463 (9)	0.0131 (8)	0.0167 (8)	0.0016 (8)
C54	0.0660 (11)	0.0510 (10)	0.0508 (9)	0.0150 (8)	0.0188 (8)	0.0104 (8)
C55	0.0496 (9)	0.0465 (9)	0.0590 (10)	0.0106 (7)	0.0165 (8)	0.0008 (8)
C56	0.0767 (13)	0.0643 (12)	0.0499 (10)	0.0217 (10)	0.0240 (9)	0.0023 (8)
C57	0.0690 (11)	0.0576 (11)	0.0558 (10)	0.0189 (9)	0.0274 (9)	0.0145 (8)
C58	0.0730 (13)	0.0476 (10)	0.0859 (14)	0.0111 (9)	0.0122 (11)	0.0092 (10)
C91	0.0463 (8)	0.0280 (7)	0.0438 (8)	−0.0020 (6)	0.0109 (7)	0.0013 (6)
C92	0.0463 (9)	0.0504 (9)	0.0530 (9)	−0.0054 (7)	0.0138 (7)	−0.0041 (7)
C93	0.0591 (10)	0.0536 (10)	0.0529 (10)	0.0009 (8)	0.0227 (8)	−0.0029 (8)
C94	0.0625 (10)	0.0364 (8)	0.0421 (8)	0.0023 (7)	0.0118 (7)	0.0010 (6)
C95	0.0475 (9)	0.0423 (9)	0.0483 (9)	−0.0042 (7)	0.0055 (7)	0.0010 (7)
C96	0.0458 (8)	0.0398 (8)	0.0461 (8)	−0.0025 (7)	0.0124 (7)	−0.0005 (7)
C97	0.0783 (14)	0.0725 (13)	0.0524 (10)	−0.0001 (10)	0.0016 (10)	−0.0124 (9)
N1	0.0498 (7)	0.0362 (7)	0.0430 (7)	0.0006 (6)	0.0050 (6)	−0.0005 (5)
N2	0.0453 (7)	0.0360 (7)	0.0499 (7)	−0.0057 (5)	0.0146 (6)	0.0011 (5)
O1	0.0837 (9)	0.0496 (7)	0.0692 (8)	0.0176 (6)	0.0235 (7)	0.0009 (6)
O2	0.0744 (9)	0.0656 (8)	0.0487 (7)	0.0045 (6)	0.0115 (6)	−0.0123 (6)
O3	0.0458 (6)	0.0555 (7)	0.0666 (7)	−0.0033 (5)	0.0162 (6)	−0.0020 (6)
O4	0.0468 (6)	0.0432 (6)	0.0806 (8)	−0.0049 (5)	0.0233 (6)	0.0039 (6)
S1	0.0707 (3)	0.0522 (3)	0.0670 (3)	0.0060 (2)	−0.0049 (2)	0.0126 (2)

### Geometric parameters (Å, º)

**Table:**

C1—N2	1.464 (2)	C18—C19	1.420 (3)
C1—C5	1.509 (2)	C18—H18	0.9300
C1—H1A	0.9700	C19—C20	1.364 (3)
C1—H1B	0.9700	C19—H19	0.9300
C2—N2	1.4578 (19)	C20—C21	1.415 (3)
C2—C3	1.533 (2)	C20—H20	0.9300
C2—H2A	0.9700	C21—C22	1.421 (3)
C2—H2B	0.9700	C22—C23	1.359 (3)
C3—C4	1.534 (2)	C22—H22	0.9300
C3—C9	1.5629 (19)	C23—C24	1.408 (3)
C3—C13	1.605 (2)	C23—H23	0.9300
C4—O4	1.2141 (18)	C24—H24	0.9300
C4—C5	1.493 (2)	C51—C52	1.466 (2)
C5—C51	1.336 (2)	C51—H51	0.9300
C6—N2	1.474 (2)	C52—C53	1.384 (2)
C6—C7	1.488 (3)	C52—C57	1.395 (2)
C6—H6A	0.9700	C53—C54	1.388 (2)
C6—H6B	0.9700	C53—H53	0.9300
C7—C8	1.290 (3)	C54—C55	1.381 (2)
C7—H7	0.9300	C54—H54	0.9300
C8—H8A	0.9300	C55—O1	1.364 (2)
C8—H8B	0.9300	C55—C56	1.390 (2)
C9—C91	1.516 (2)	C56—C57	1.372 (3)
C9—C10	1.523 (2)	C56—H56	0.9300
C9—H9	0.9800	C57—H57	0.9300
C10—N1	1.4533 (19)	C58—O1	1.422 (2)
C10—C11	1.521 (2)	C58—H58A	0.9600
C10—H10	0.9800	C58—H58B	0.9600
C11—S1	1.8349 (18)	C58—H58C	0.9600
C11—H11A	0.9700	C91—C96	1.384 (2)
C11—H11B	0.9700	C91—C92	1.392 (2)
C12—N1	1.447 (2)	C92—C93	1.384 (2)
C12—S1	1.8285 (18)	C92—H92	0.9300
C12—H12A	0.9700	C93—C94	1.376 (2)
C12—H12B	0.9700	C93—H93	0.9300
C13—N1	1.4654 (19)	C94—C95	1.376 (2)
C13—C17	1.525 (2)	C94—O2	1.3782 (19)
C13—C14	1.578 (2)	C95—C96	1.395 (2)
C14—O3	1.2145 (19)	C95—H95	0.9300
C14—C15	1.482 (2)	C96—H96	0.9300
C15—C24	1.370 (2)	C97—O2	1.418 (2)
C15—C16	1.402 (2)	C97—H97A	0.9600
C16—C21	1.403 (2)	C97—H97B	0.9600
C16—C17	1.409 (2)	C97—H97C	0.9600
C17—C18	1.361 (2)		
			
N2—C1—C5	111.93 (12)	C19—C18—H18	120.6
N2—C1—H1A	109.2	C20—C19—C18	122.58 (19)
C5—C1—H1A	109.2	C20—C19—H19	118.7
N2—C1—H1B	109.2	C18—C19—H19	118.7
C5—C1—H1B	109.2	C19—C20—C21	120.13 (17)
H1A—C1—H1B	107.9	C19—C20—H20	119.9
N2—C2—C3	108.17 (12)	C21—C20—H20	119.9
N2—C2—H2A	110.1	C16—C21—C20	116.11 (17)
C3—C2—H2A	110.1	C16—C21—C22	115.39 (19)
N2—C2—H2B	110.1	C20—C21—C22	128.50 (18)
C3—C2—H2B	110.1	C23—C22—C21	121.18 (18)
H2A—C2—H2B	108.4	C23—C22—H22	119.4
C2—C3—C4	106.66 (12)	C21—C22—H22	119.4
C2—C3—C9	113.43 (12)	C22—C23—C24	122.47 (18)
C4—C3—C9	111.42 (12)	C22—C23—H23	118.8
C2—C3—C13	110.74 (11)	C24—C23—H23	118.8
C4—C3—C13	110.21 (11)	C15—C24—C23	117.92 (19)
C9—C3—C13	104.43 (11)	C15—C24—H24	121.0
O4—C4—C5	121.71 (14)	C23—C24—H24	121.0
O4—C4—C3	121.59 (13)	C5—C51—C52	129.37 (15)
C5—C4—C3	116.69 (12)	C5—C51—H51	115.3
C51—C5—C4	116.29 (14)	C52—C51—H51	115.3
C51—C5—C1	124.63 (14)	C53—C52—C57	117.24 (15)
C4—C5—C1	118.97 (13)	C53—C52—C51	124.48 (15)
N2—C6—C7	115.66 (14)	C57—C52—C51	118.27 (15)
N2—C6—H6A	108.4	C52—C53—C54	121.87 (16)
C7—C6—H6A	108.4	C52—C53—H53	119.1
N2—C6—H6B	108.4	C54—C53—H53	119.1
C7—C6—H6B	108.4	C55—C54—C53	119.58 (16)
H6A—C6—H6B	107.4	C55—C54—H54	120.2
C8—C7—C6	126.7 (2)	C53—C54—H54	120.2
C8—C7—H7	116.6	O1—C55—C54	125.01 (16)
C6—C7—H7	116.6	O1—C55—C56	115.43 (15)
C7—C8—H8A	120.0	C54—C55—C56	119.55 (16)
C7—C8—H8B	120.0	C57—C56—C55	119.94 (16)
H8A—C8—H8B	120.0	C57—C56—H56	120.0
C91—C9—C10	116.94 (12)	C55—C56—H56	120.0
C91—C9—C3	116.10 (12)	C56—C57—C52	121.78 (16)
C10—C9—C3	102.54 (11)	C56—C57—H57	119.1
C91—C9—H9	106.9	C52—C57—H57	119.1
C10—C9—H9	106.9	O1—C58—H58A	109.5
C3—C9—H9	106.9	O1—C58—H58B	109.5
N1—C10—C11	105.13 (12)	H58A—C58—H58B	109.5
N1—C10—C9	100.42 (12)	O1—C58—H58C	109.5
C11—C10—C9	118.34 (13)	H58A—C58—H58C	109.5
N1—C10—H10	110.7	H58B—C58—H58C	109.5
C11—C10—H10	110.7	C96—C91—C92	117.19 (14)
C9—C10—H10	110.7	C96—C91—C9	119.82 (13)
C10—C11—S1	104.91 (11)	C92—C91—C9	122.99 (14)
C10—C11—H11A	110.8	C93—C92—C91	121.24 (16)
S1—C11—H11A	110.8	C93—C92—H92	119.4
C10—C11—H11B	110.8	C91—C92—H92	119.4
S1—C11—H11B	110.8	C94—C93—C92	120.59 (16)
H11A—C11—H11B	108.8	C94—C93—H93	119.7
N1—C12—S1	102.73 (11)	C92—C93—H93	119.7
N1—C12—H12A	111.2	C93—C94—C95	119.52 (15)
S1—C12—H12A	111.2	C93—C94—O2	115.79 (15)
N1—C12—H12B	111.2	C95—C94—O2	124.68 (16)
S1—C12—H12B	111.2	C94—C95—C96	119.57 (15)
H12A—C12—H12B	109.1	C94—C95—H95	120.2
N1—C13—C17	111.04 (12)	C96—C95—H95	120.2
N1—C13—C14	113.34 (12)	C91—C96—C95	121.90 (15)
C17—C13—C14	101.65 (12)	C91—C96—H96	119.1
N1—C13—C3	101.45 (11)	C95—C96—H96	119.1
C17—C13—C3	117.25 (12)	O2—C97—H97A	109.5
C14—C13—C3	112.61 (12)	O2—C97—H97B	109.5
O3—C14—C15	127.24 (15)	H97A—C97—H97B	109.5
O3—C14—C13	124.21 (14)	O2—C97—H97C	109.5
C15—C14—C13	107.82 (13)	H97A—C97—H97C	109.5
C24—C15—C16	119.92 (16)	H97B—C97—H97C	109.5
C24—C15—C14	132.45 (17)	C12—N1—C10	109.96 (13)
C16—C15—C14	107.60 (13)	C12—N1—C13	121.79 (12)
C15—C16—C21	123.09 (16)	C10—N1—C13	109.94 (12)
C15—C16—C17	113.08 (14)	C2—N2—C1	111.62 (12)
C21—C16—C17	123.81 (17)	C2—N2—C6	113.38 (12)
C18—C17—C16	118.48 (15)	C1—N2—C6	112.06 (12)
C18—C17—C13	131.99 (15)	C55—O1—C58	118.67 (15)
C16—C17—C13	109.53 (14)	C94—O2—C97	116.86 (14)
C17—C18—C19	118.88 (17)	C12—S1—C11	93.49 (7)
C17—C18—H18	120.6		
			
N2—C2—C3—C4	−63.55 (14)	C15—C16—C21—C20	177.70 (15)
N2—C2—C3—C9	173.41 (12)	C17—C16—C21—C20	−0.5 (2)
N2—C2—C3—C13	56.38 (15)	C15—C16—C21—C22	−1.9 (2)
C2—C3—C4—O4	−139.41 (15)	C17—C16—C21—C22	179.93 (15)
C9—C3—C4—O4	−15.1 (2)	C19—C20—C21—C16	−0.6 (3)
C13—C3—C4—O4	100.31 (16)	C19—C20—C21—C22	178.93 (19)
C2—C3—C4—C5	41.65 (17)	C16—C21—C22—C23	0.6 (3)
C9—C3—C4—C5	165.93 (12)	C20—C21—C22—C23	−178.87 (19)
C13—C3—C4—C5	−78.62 (15)	C21—C22—C23—C24	1.1 (3)
O4—C4—C5—C51	−28.6 (2)	C16—C15—C24—C23	0.4 (2)
C3—C4—C5—C51	150.36 (14)	C14—C15—C24—C23	177.97 (17)
O4—C4—C5—C1	155.09 (15)	C22—C23—C24—C15	−1.6 (3)
C3—C4—C5—C1	−26.0 (2)	C4—C5—C51—C52	−176.30 (16)
N2—C1—C5—C51	−147.40 (16)	C1—C5—C51—C52	−0.2 (3)
N2—C1—C5—C4	28.6 (2)	C5—C51—C52—C53	−41.8 (3)
N2—C6—C7—C8	−127.7 (2)	C5—C51—C52—C57	139.11 (19)
C2—C3—C9—C91	33.48 (17)	C57—C52—C53—C54	−1.0 (3)
C4—C3—C9—C91	−86.90 (15)	C51—C52—C53—C54	179.92 (17)
C13—C3—C9—C91	154.14 (12)	C52—C53—C54—C55	−0.8 (3)
C2—C3—C9—C10	−95.26 (14)	C53—C54—C55—O1	−178.99 (17)
C4—C3—C9—C10	144.36 (12)	C53—C54—C55—C56	1.5 (3)
C13—C3—C9—C10	25.40 (14)	O1—C55—C56—C57	179.99 (18)
C91—C9—C10—N1	−170.48 (12)	C54—C55—C56—C57	−0.5 (3)
C3—C9—C10—N1	−42.27 (13)	C55—C56—C57—C52	−1.4 (3)
C91—C9—C10—C11	75.89 (18)	C53—C52—C57—C56	2.1 (3)
C3—C9—C10—C11	−155.90 (13)	C51—C52—C57—C56	−178.78 (18)
N1—C10—C11—S1	33.74 (15)	C10—C9—C91—C96	−145.25 (14)
C9—C10—C11—S1	144.77 (12)	C3—C9—C91—C96	93.40 (16)
C2—C3—C13—N1	123.33 (12)	C10—C9—C91—C92	33.9 (2)
C4—C3—C13—N1	−118.89 (12)	C3—C9—C91—C92	−87.40 (18)
C9—C3—C13—N1	0.89 (14)	C96—C91—C92—C93	0.7 (2)
C2—C3—C13—C17	−115.57 (14)	C9—C91—C92—C93	−178.55 (15)
C4—C3—C13—C17	2.21 (17)	C91—C92—C93—C94	−0.4 (3)
C9—C3—C13—C17	121.99 (13)	C92—C93—C94—C95	−0.5 (3)
C2—C3—C13—C14	1.85 (17)	C92—C93—C94—O2	178.49 (15)
C4—C3—C13—C14	119.63 (13)	C93—C94—C95—C96	1.1 (2)
C9—C3—C13—C14	−120.59 (13)	O2—C94—C95—C96	−177.86 (15)
N1—C13—C14—O3	−46.1 (2)	C92—C91—C96—C95	−0.1 (2)
C17—C13—C14—O3	−165.29 (15)	C9—C91—C96—C95	179.13 (14)
C3—C13—C14—O3	68.39 (19)	C94—C95—C96—C91	−0.8 (2)
N1—C13—C14—C15	124.83 (13)	S1—C12—N1—C10	45.17 (15)
C17—C13—C14—C15	5.60 (15)	S1—C12—N1—C13	175.85 (11)
C3—C13—C14—C15	−120.72 (13)	C11—C10—N1—C12	−53.36 (16)
O3—C14—C15—C24	−11.9 (3)	C9—C10—N1—C12	−176.71 (12)
C13—C14—C15—C24	177.56 (17)	C11—C10—N1—C13	169.93 (12)
O3—C14—C15—C16	165.89 (16)	C9—C10—N1—C13	46.58 (14)
C13—C14—C15—C16	−4.64 (17)	C17—C13—N1—C12	74.54 (18)
C24—C15—C16—C21	1.4 (2)	C14—C13—N1—C12	−39.15 (19)
C14—C15—C16—C21	−176.74 (14)	C3—C13—N1—C12	−160.12 (13)
C24—C15—C16—C17	179.76 (15)	C17—C13—N1—C10	−154.78 (12)
C14—C15—C16—C17	1.63 (18)	C14—C13—N1—C10	91.54 (14)
C15—C16—C17—C18	−177.11 (15)	C3—C13—N1—C10	−29.43 (15)
C21—C16—C17—C18	1.2 (2)	C3—C2—N2—C1	71.71 (15)
C15—C16—C17—C13	2.21 (18)	C3—C2—N2—C6	−160.60 (13)
C21—C16—C17—C13	−179.43 (14)	C5—C1—N2—C2	−51.34 (17)
N1—C13—C17—C18	53.6 (2)	C5—C1—N2—C6	−179.74 (13)
C14—C13—C17—C18	174.48 (17)	C7—C6—N2—C2	−61.56 (19)
C3—C13—C17—C18	−62.3 (2)	C7—C6—N2—C1	65.90 (19)
N1—C13—C17—C16	−125.56 (13)	C54—C55—O1—C58	−6.9 (3)
C14—C13—C17—C16	−4.72 (16)	C56—C55—O1—C58	172.63 (17)
C3—C13—C17—C16	118.49 (14)	C93—C94—O2—C97	172.07 (16)
C16—C17—C18—C19	−0.9 (2)	C95—C94—O2—C97	−9.0 (2)
C13—C17—C18—C19	179.97 (16)	N1—C12—S1—C11	−19.62 (13)
C17—C18—C19—C20	−0.2 (3)	C10—C11—S1—C12	−8.01 (13)
C18—C19—C20—C21	0.9 (3)		

### Hydrogen-bond geometry (Å, º)

*Cg*1 is the centroid of the C52–C57 ring.

**Table:**

*D*—H···*A*	*D*—H	H···*A*	*D*···*A*	*D*—H···*A*
C2—H2*B*···O3	0.97	2.45	2.9587 (19)	113
C93—H93···O1^i^	0.93	2.49	3.343 (2)	153
C58—H58*B*···*Cg*1^ii^	0.96	2.85	3.487 (2)	125

Symmetry codes: (i) *x*−1/2, −*y*+1/2, *z*+1/2; (ii) −*x*+1, −*y*, −*z*+1.
